# *BRAF*^V600E^ mutation in anaplastic thyroid carcinomas and their accompanying differentiated carcinomas

**DOI:** 10.1038/sj.bjc.6603764

**Published:** 2007-04-24

**Authors:** T Takano, Y Ito, M Hirokawa, H Yoshida, A Miyauchi

**Affiliations:** 1 Department of Laboratory Medicine, Osaka University Graduate School of Medicine, D2, 2-2 Yamadaoka, Suita, Osaka 565-0871, Japan; 2 Kuma Hospital, 8-2-35, Simoyamate-Dori, Chuo-Ku, Kobe, Hyogo 650-0011, Japan

**Keywords:** thyroid, anaplastic carcinoma, papillary carcinoma, *BRAF*, mutation

## Abstract

Frequency of a *BRAF*^V600E^ mutation in anaplastic thyroid carcinoma, which is thought to be derived mainly from papillary carcinoma by multi-step carcinogenesis, is much lower than that in papillary carcinomas. To clarify this phenomenon, we analysed *BRAF*^V600E^ mutation in 20 cases of anaplastic carcinoma and 13 accompanying differentiated carcinomas. Among twenty cases of anaplastic carcinomas, nine and four accompanied papillary and follicular carcinomas, respectively. *BRAF*^V600E^ mutation was found in four (20%) cases. *BRAF*^V600E^ mutation was found in three of nine (33.3%), none of four and one of seven (14.3%) anaplastic carcinomas with papillary carcinoma, follicular carcinoma and without differentiated components, respectively. All three papillary carcinomas accompanied by anaplastic carcinoma with a *BRAF*^V600E^ mutation were also shown to have a *BRAF*^V600E^ mutation. In summary, *BRAF*^V600E^ mutation was occasionally observed in anaplastic carcinomas with papillary carcinoma, and the low frequency of *BRAF*^V600E^ mutation in anaplastic carcinoma was thought to be due to the low frequency of anaplastic carcinomas with papillary carcinoma. These findings raise a question about the classical model of anaplastic transformation and suggest some roles of thyroid cancer stem cells in the generation of anaplastic carcinoma.

Thyroid carcinomas are thought to be generated from normal thyroid follicular cells (thyrocytes) by multi-step carcinogenesis (Farid *et al*, 1994). According to this hypothesis, anaplastic carcinomas are generated from both follicular and papillary carcinomas by genomic changes, such as mutations in *TP53*. Follicular carcinomas are generated from follicular adenomas, while papillary carcinomas are derived from some unknown precursor cells that are generated from normal thyrocytes. Recently, a somatic point mutation in the *BRAF* gene has been identified as the most common genetic event in papillary thyroid carcinoma (Kimura *et al*, 2003). Most of the tumours with a mutation harboured a thymine-to-adenine transversion at nucleotide position 1799, which results in a valine-to-glutamic acid substitution at residue 600 (V600E). Previous reports indicated that *BRAF* mutations in thyroid tumours are generally restricted to papillary carcinoma, and usually there is no mutation in other types of well-differentiated thyroid cancers, including follicular carcinoma, Hürthle carcinoma and medullary carcinoma, as well as in benign thyroid tumours. The frequency of the mutations in papillary carcinomas is ranged from 29 to 83% of papillary carcinoma (Xing, 2005).

It remains controversial whether this gene is also mutated in anaplastic thyroid carcinomas. In iodine-sufficient areas such as Japan, papillary carcinoma accounts for 90% of thyroid differentiated carcinomas and follicular carcinoma is rare (Hay, 1990). When multi-step carcinogenesis is taken into account, a considerable number of anaplastic carcinomas with *BRAF* mutations should be found. In previous reports, however, the frequency of the *BRAF* mutation was only about 10% on average and ranged from 0 to 63% (Fukushima *et al*, 2003; Namba *et al*, 2003; Nikiforova *et al*, 2003; Begum *et al*, 2004; Soares *et al*, 2004; Xing *et al*, 2004; Quiros *et al*, 2005). Among these studies, some reported that the *BRAF* mutation is found frequently only in anaplastic carcinomas with a papillary carcinoma component, although these studies have examined only four or five cases. A study examining a larger number of samples is essential to clarify the role of the *BRAF* mutation in anaplastic carcinoma.

Considering these facts, we examined *BRAF*^V600E^ mutation in 20 anaplastic carcinomas and 13 accompanying differentiated carcinomas and found a low frequency of *BRAF*^V600E^ mutation in anaplastic carcinomas due to the low frequency of anaplastic carcinomas accompanied by papillary carcinoma.

## MATERIALS AND METHODS

Twenty anaplastic carcinomas were used in this study. Among these cases, nine accompanied papillary carcinoma and four accompanied follicular carcinoma. Twenty papillary carcinomas without an undifferentiated component were also examined. These tumours were surgically resected at Kuma Hospital after obtaining the patient's informed consent. The experimental protocol was approved by the local ethical committee. Paraffin-embedded tissues were microdissected to separately study well-differentiated and anaplastic areas within the same tumour nodule. For microdissection, 8 *μ*m sections from a paraffin-embedded tissue were put on glass slides and weakly stained with haematoxylin and eosin. The areas of interest (approximately up to 0.5 cm^2^) were microdissected using the AS LMD (Leica, Tokyo, Japan). Genomic DNA was isolated using a QIAamp DNA Mini Kit (Qiagen, Tokyo, Japan). Ten microlitres of DNA extracted from each tumour sample was examined by direct sequencing after amplification by polymerase chain reaction (PCR). To avoid carryover contamination, Platinum Quantitative PCR SuperMix-UDG (Invitrogen Japan, Tokyo, Japan) was used for PCR amplification. Fifty microlitres of PCR mixture contained 0.5 *μ*M of each primer, 1 *μ*l of 50 mM magnesium chloride, 10 *μ*l of extracted DNA and 25 *μ*l of Platinum Quantitative PCR SuperMix-UDG. The primers used were

BRF: 5′-ACTCTTCATAATGCTTGCTCTGATAG -3′ and

BRR: 5′-CTGATGGGACCCACTCCA -3′

The PCR conditions were 50°C for 2 min, 95°C for 2 min and 40 cycles of 95°C for 15 s and 60°C for 1 min. The PCR products were separated on agarose gel and extracted with a QIAquick Gel Extraction Kit (Qiagen). Purified fragments were sequenced using a BigDye Terminator Cycle Sequencing FS Ready Reaction Kit with an ABI PRISM 310 Genetic Analyzer (Applied Biosystems, Tokyo, Japan).

## RESULTS

The results of the mutation analysis are summarised in Tables 1 and 2. *BRAF*^V600E^ mutation was found in 4 (20%) of 20 anaplastic carcinomas and in 9 (45%) of 20 papillary carcinomas without an anaplastic carcinoma examined as controls.

Among the 20 cases of anaplastic carcinomas, 9 and 4 were accompanied by papillary and follicular carcinomas, respectively. *BRAF*^V600E^ mutation was found in three of nine (33.3%) anaplastic carcinomas accompanied by papillary carcinoma, none of four anaplastic carcinomas accompanied by follicular carcinoma and one of seven (14.3%) anaplastic carcinomas without differentiated components. All three papillary carcinomas accompanied by anaplastic carcinoma with a *BRAF*^V600E^ mutation were also shown to have a *BRAF*^V600E^ mutation, whereas there was no *BRAF*^V600E^ mutation in follicular carcinomas with anaplastic carcinoma.

**Figure 1:**
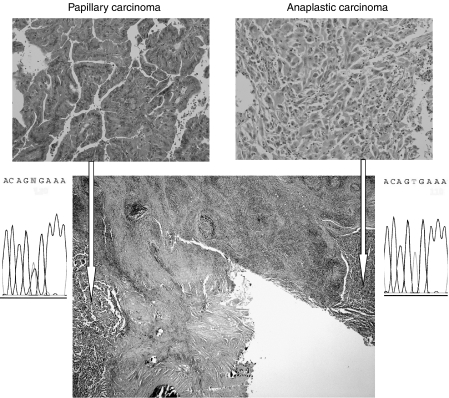
Haematoxylin and eosin-stained section and the results of sequencing analysis in case 9. Upper and lower panels show high (× 200) and low magnification of a section, respectively. Areas indicated by white arrows were microdissected and the sequence of the *BRAF* gene was analysed. A heterozygous missense mutation (T1799A/V600E) was identified in exon 15 in a papillary carcinoma sample (left) but not in a coexisting anaplastic carcinoma sample (left).

In case 9, a *BRAF*^V600E^ mutation was found only in papillary carcinoma but not in coexisting anaplastic carcinoma (Figure 1). However, whether these two tumours were derived from the same precursor was not clear, since a thick connective tissue separated the two.

## DISCUSSION

The results of previous studies on the frequency of a *BRAF*^V599R^ mutation in papillary and anaplastic carcinomas in the Japanese population are summarised in Table 3. The frequency of a *BRAF*^V599R^ mutation in anaplastic carcinomas is about half of that in papillary carcinomas. It has generally been thought that most anaplastic carcinomas are derived from papillary carcinomas by accumulation of genomic damage, but if this hypothesis is true, then the present observations are difficult to explain. One possible explanation is that papillary carcinoma with *BRAF*^V599R^ mutation shows a better prognosis without proceeding to anaplastic transformation. However, this theory conflicts with some previous studies showing papillary carcinomas with *BRAF*^V599R^ mutation show a poor prognosis (Xing *et al*, 2005). Another explanation is that follicular carcinomas are more likely to transform into an anaplastic carcinoma than papillary carcinoma. In fact, nine cases of anaplastic carcinoma with a papillary component showed a high frequency of *BRAF*^V599R^ mutation. This might be partly true, since even though both follicular and anaplastic carcinomas are rare in iodine-sufficient countries like Japan, many cases of anaplastic carcinoma accompanying a follicular carcinoma have been reported (Yokozawa *et al*, 1996; Asakawa and Kobayashi, 2002). However, in our study, there were only four cases of anaplastic carcinomas with a follicular carcinoma, which is not sufficient to explain the decreased frequency. Seven of twenty anaplastic carcinomas did not demonstrate any differentiated components and *BRAF*^V599R^ mutation was detected in only one of these cases. This seems to be the major cause of the decreased frequency of a *BRAF*^V599R^ mutation in anaplastic carcinomas.

The origin of anaplastic carcinoma is quite puzzling. In multi-step carcinogenesis, malignant transformation is caused by the accumulation of genomic damage in cancer cells. It is thought that anaplastic carcinoma arises by malignant transformation of coexisting papillary carcinoma. However, our present findings indicate that papillary carcinoma with *BRAF* mutation, which comprises a considerable percentage of differentiated thyroid carcinomas, might not be the major origin of anaplastic carcinoma. Similar phenomena are observed in other genes. Rearrangement of the *RET* and *PAX8-PPARγ1* gene is found frequently in papillary and follicular carcinomas, respectively, but not in anaplastic carcinomas (Tallini *et al*, 1998; Kroll *et al*, 2000).

Considering these discrepancies, a new hypothesis of thyroid carcinogenesis, fetal cell carcinogenesis, in which cancer cells are derived from the remnants of fetal thyroid cells, instead of normal thyroid follicular cells, has been presented (Takano, 2004, 2007; Takano and Amino, 2005). In this hypothesis, the origins of anaplastic carcinoma are not differentiated carcinoma cells but certain kinds of fetal thyroid cells, possibly cells with a close relationship with thyroid stem cells. It is noted that in this hypothesis, the role of *BRAF* mutation is the prevention of immature fetal thyroid cells, namely thyroblasts that are the origins of papillary carcinomas, from differentiating into follicle-forming cells, such as follicular tumour cells or thyrocytes.

**Figure 2:**
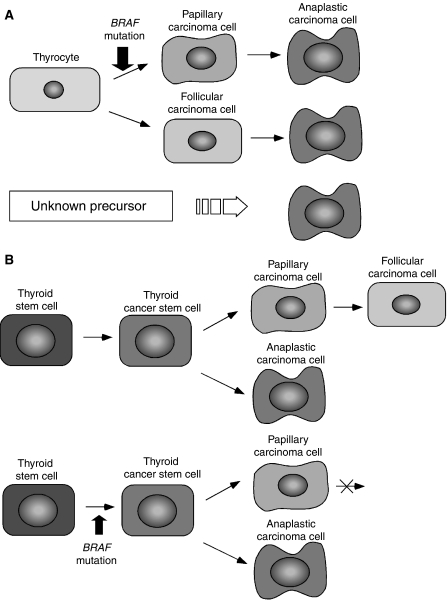
Anaplastic transformation in multi-step carcinogenesis and fetal cell carcinogenesis. In multi-step carcinogenesis (**A**), anaplastic carcinoma cells are generated by three pathways. A thyrocyte is transformed into a papillary carcinoma cell by a *BRAF* mutation, then further transformed into an anaplastic carcinoma cell. A thyrocyte without a *BRAF* mutation is transformed into a follicular carcinoma cells, then further transformed into an anaplastic carcinoma cell. Anaplastic carcinomas are also generated from some unknown precursors. In fetal cell carcinogenesis (**B**), both anaplastic and differentiated carcinoma cells are generated from thyroid cancer stem cells. A thyroid cancer stem cell with a *BRAF* mutation can generate anaplastic or papillary carcinoma cells but not follicular carcinoma cells, since a *BRAF* mutation blocks the papillary carcinoma cell from differentiating into a follicular carcinoma cell.

In multi-step carcinogenesis (Figure 2), the present data are explained as follows. A thyrocyte is transformed into a papillary carcinoma cell by a *BRAF* mutation and it is further transformed into an anaplastic carcinoma cell by a *TP53* mutation. A thyrocyte without *BRAF* mutation is transformed into a follicular carcinoma cells and is further transformed into an anaplastic carcinoma cell by a *TP53* mutation. A considerable number of anaplastic carcinomas are derived from some unknown precursors, which remain silent for many years without proliferation.

In fetal cell carcinogenesis, the present data are explained as follows. Both anaplastic carcinoma cells and differentiated carcinomas cells are derived from the same origin, probably thyroid cancer stem cells. In other words, thyroid cancer stem cells can produce either anaplastic carcinoma cells or differentiated cancer cells. Since a *BRAF* mutation blocks a papillary carcinoma cell from differentiating into follicular cells, a thyroid cancer stem cell with *BRAF* mutation produces anaplastic carcinoma cells and papillary carcinoma cells, but not follicular carcinoma cells. On the contrary, thyroid cancer stem cells without *BRAF* mutation produce anaplastic carcinoma cells and follicular carcinoma cells, since the papillary carcinoma cells that were produced further differentiate immediately into follicular cells. Occasionally, thyroid cancer stem cells maintain an undifferentiated property and proliferate without producing differentiated carcinoma cells, resulting in the formation of anaplastic carcinoma without differentiated components. Late onset of anaplastic carcinoma is easily understood when the origin of anaplastic carcinoma is a thyroid stem cell, since a stem cell can stay silent without proliferation for many years (Reya *et al*, 2001). Similarly, the possibility that thyroid cancer stem cells are the unknown origin of anaplastic carcinoma might also be taken into consideration in multi-step carcinogenesis.

It will be a direct proof of fetal cell carcinogenesis hypothesis when a *BRAF* mutation is observed only in coexisting papillary carcinoma but not in anaplastic carcinoma. Case 9 is such a case. However, the relation of these two tumours is not clear, since they were separated by a connective tissue. At least in this case, anaplastic carcinoma is not derived from coexisting papillary carcinoma but from an unknown origin. Analysing more cases in the same way may lead to the finding of similar cases with discrepant genetic alternation, since a relatively small number of cases were engaged in our and previous studies, due to the rarity of anaplastic carcinoma.

Although the mechanism of anaplastic transformation has not been understood, the recent advances in cancer research, the elucidation of cancer stem cells, may provide new perspectives that will contribute to clarifying the nature of anaplastic carcinoma. The present study may facilitate an understanding of the relation between anaplastic carcinoma and coexisting differentiated carcinomas.

## Figures and Tables

**Table 1:** *BRAF*^V600E^ mutation in anaplastic carcinomas and their accompanying differentiated carcinomas

**No.**	**Gender**	**Age**	**Accompanying DC**	***BRAF*^V600E^ AC**	**Mutation in DC**
1	Male	48	Papillary carcinoma	+	+
2	Female	63	Papillary carcinoma	−	−
3	Female	80	Papillary carcinoma	+	+
4	Female	59	Papillary carcinoma	−	−
5	Female	88	Papillary carcinoma	+	+
6	Female	64	Papillary carcinoma	−	−
7	Male	52	Papillary carcinoma	−	−
8	Female	65	Papillary carcinoma	−	−
9	Female	75	Papillary carcinoma	−	+
10	Female	35	Follicular carcinoma	−	−
11	Female	61	Follicular carcinoma	−	−
12	Female	57	Follicular carcinoma	−	−
13	Female	62	Follicular carcinoma	−	−
14	Female	77	—	−	
15	Female	63	—	−	
16	Female	80	—	−	
17	Female	82	—	−	
18	Female	77	—	+	
19	Female	54	—	−	
20	Female	85	—	−	

AC=anaplastic carcinoma; DC=differentiated carcinoma.

**Table 2:** Summary of the *BRAF*^V600E^ mutation in anaplastic carcinomas and their accompanying differentiated carcinomas

**Histology**	**Total number**	**Mutation (+)**	**Mutation (−)**
All anaplastic carcinomas	20	4 (20%)	16 (80%)
Anaplastic carcinoma with papillary carcinoma	9	3 (33.3%)	6 (66.7%)
Anaplastic carcinoma with follicular carcinoma	4	0 (0%)	4 (100%)
Anaplastic carcinoma without differentiated carcinoma	7	1 (14.3%)	6 (85.7%)
Papillary carcinoma with anaplastic carcinoma	9	4 (44.4%)	5 (55.6%)
Follicular carcinoma with anaplastic carcinoma	4	0 (0%)	4 (100%)
Papillary carcinoma without anaplastic carcinoma	20	9 (45%)	11 (55%)

**Table 3:** *BRAF*^V600E^ mutation in papillary and anaplastic carcinomas in the Japanese population

	**Papillary carcinoma**		**Anaplastic carcinoma**	
**Reference**	**Positive/total cases**	**%**	**Positive/total cases**	**%**
Fukushima *et al* (2003)	40/76	52.6	0/7	0
Namba *et al* (2003)	49/170	28.8	2/6	33.3
This study	9/20	45.0	4/20	20.0
Total	98/266	36.8	6/33	18.2
